# Yokukansan Inhibits the Development of Morphine Tolerance by Regulating Presynaptic Proteins in DRG Neurons

**DOI:** 10.3389/fphar.2022.862539

**Published:** 2022-05-18

**Authors:** Yusuke Ohashi, Fatma Zahra Sakhri, Hideshi Ikemoto, Takayuki Okumo, Naoki Adachi, Masataka Sunagawa

**Affiliations:** Department of Physiology, School of Medicine, Showa University, Tokyo, Japan

**Keywords:** morphine tolerance, pain management, traditional medicine, kampo medicine, spinal cord, yokukansan

## Abstract

Opioids, such as morphine, are used in clinical settings for the management of acute and chronic pain. However, long-term use of morphine leads to antinociceptive tolerance and hypersensitivity. The cellular and molecular mechanisms of morphine tolerance seem to be quite complex, with suggestions including internalization of the μ-opioid receptor (MOR), neuroinflammation with activation of microglia and astrocytes, and changes in synaptic function in the central nervous system. Yokukansan (YKS), a traditional Kampo medicine consisting of seven herbs, has been used to treat emotional instability, neurosis, and insomnia. Interestingly, recent studies have begun to reveal the inhibitory effect of YKS on the development of morphine tolerance. In the present study, we determined the effect of YKS on morphine tolerance formation and its mechanisms in a rat model, focusing on the synapses between primary sensory neurons and spinal dorsal horn secondary neurons. We found that morphine tolerance formation was significantly inhibited by YKS (0.3 or 1.0 g/kg/day) preadministration for 7 days. Repeated administration of morphine (10 mg/kg/day) increased the expression of presynaptic proteins, including synaptotagmin I, in the spinal cord, which was suppressed by YKS. Furthermore, these changes in presynaptic protein expression were more pronounced at isolectin B4 (IB4)-positive excitatory synapses around the lamina II of the dorsal horn. These results suggest that YKS suppresses the development of morphine tolerance by inhibiting the enhancement of presynaptic function of dorsal root ganglia neurons projecting to spinal dorsal horn neurons caused by continuous morphine administration.

## Introduction

Opioids, such as morphine and fentanyl, remain the gold standard for acute and chronic pain in the clinical setting. However, prolonged use of morphine in pain management reduces efficacy and increases sensitivity, which is called “morphine tolerance”. The development of morphine tolerance limits the use of morphine in patients who require long-term morphine administration. For decades, several cellular and molecular mechanisms have been suggested to be involved in morphine tolerance, including internalization of the μ-opioid receptors (MORs) (Williams et al., 2013), neuroinflammation through activation of microglia and astrocytes (Raghavendra et al., 2002; Roeckel et al., 2016), and regulation of N-methyl-D-aspartate (NMDA) receptors expression or functions (Price et al., 2000; Sandkühler, 2009), in the central nervous system.

Recent studies have shown that opioids alter the properties of neurons expressing MORs and nociceptive circuits at the level of the dorsal root ganglia (DRG) and spinal cord dorsal horn (Christie, 2008; Rivat and Ballantyne, 2016). Surprisingly, in mice lacking MORs in the transient receptor potential channel vanilloid type 1 (TRPV1)-expressing DRG neurons, nociceptor-specific deletion of MORs, abolished morphine tolerance and opioid-induced hyperalgesia without altering antinociception (Corder et al., 2017), and MORs ablation in all populations of primary sensory neurons in the DRG diminishes both opioid-induced analgesic effects and hyperalgesia (Sun et al., 2020). Furthermore, Zhou et al. showed that opioid-induced hyperalgesia is attributed to potentiation of glutamatergic synaptic transmission between DRG neurons and the secondary neurons in the spinal cord dorsal horn, which is dependent on presynaptic changes (Zhou et al., 2010).

Kampo medicine, used in traditional Japanese medicine, has been used to treat a variety of pains, with or without Western medicine (Arai et al., 2020; Sunagawa et al., 2021). Yokukansan (YKS; Yi-Gan San in Chinese), which was firstly described in the Bao-ying jin-jing-lu written in 1,550, is a traditional Kampo medicine consisting of seven herbs. YKS is used to treat emotional irritability, neurosis, and insomnia. Recently, it has been reported that morphine tolerance in experimental animals is weakened when YKS is administered daily for 3 weeks prior to the treatment (Nakagawa et al., 2012). It has also been reported that YKS suppresses the development of morphine tolerance by inhibiting the secretion of orexin A in the midbrain (Katayama et al., 2018) and spinal glial activation (Takemoto et al., 2016) in rats. Thus, various mechanisms were considered to be involved in the effect of YKS on the development of morphine tolerance.

In the present study, therefore, using an animal model of morphine tolerance, we investigated the inhibitory effect of YKS on the development of morphine tolerance, focusing on the synapses between DRG neurons and secondary neurons in the spinal cord dorsal horn.

## Materials and Methods

### Animals

Wistar rats (male, 7 weeks, around 245–255 g) were purchased from Nippon Bio-Supp. Center (Tokyo, Japan) and housed three or four per cage in 12:12 h light/dark in our animal facilities at 25 ± 1°C, with 45 ± 5% humidity, and free access to food (CLEA Japan, CE-2, Tokyo, Japan) and water for 7 days prior to the experiment. Each experiment was repeated two or three times and all efforts were made to minimize animal suffering and to reduce the number of animals used.

This study was conducted with the approval of the Committee of Animal Care and Welfare of Showa University and in accordance with the guidelines established by the Committee of Animal Care and Welfare of Showa University (certificate number: 03072; date of approval: 1 April 2020).

### Drug Administration

**FIGURE 1:**
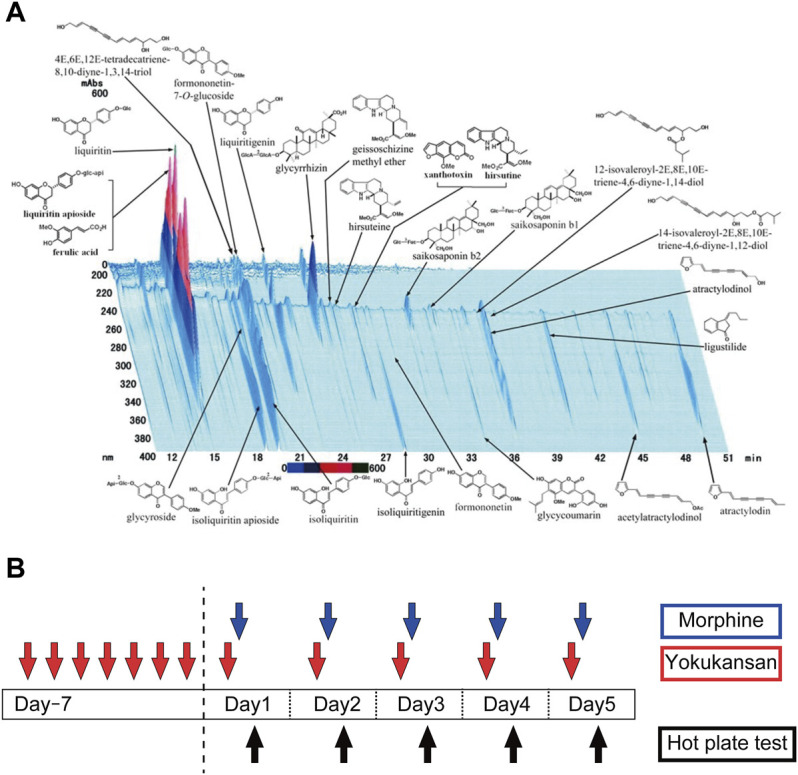
**(A)** Constituents of the YKS extract identified by 3D-HPLC (provided by Tsumura and Co.). See details (Ikarashi and Mizoguchi, 2016) **(B)** The experimental schedule to develop a morphine antinociceptive tolerance rat model and to clarify the inhibitory effect of YKS on morphine tolerance. Morphine tolerant model rats were developed by subcutaneous injection of morphine (10 mg/kg/day) daily for 5 days and oral administration of YKS (0.3 g/kg or 1.0 g/kg/day) started 7 days before the first morphine injection. Morphine was injected 30 min after YKS administration. The hot plate test was performed 1 h after morphine injection. Spinal cord samples for Western blotting and immunohistochemistry were collected on Day 5.

The dry powdered extract of YKS (Lot No. 2110054010) was supplied by Tsumura Corporation (Japan). The seven herbs, *Atractylodes lancea* (Thunb.) DC [Asteraceae] (4.0 g), *Smilax glabra* Roxb [Smilacaceae] (4.0 g), *Conioselinum anthriscoides* (H.Boissieu) Pimenov and Kljuykov ‘Chuanxiong’ [Apiaceae] (3.0 g), *Uncaria rhynchophylla* (Miq.) Miq [Rubiaceae] (3.0 g), *Angelica sinensis* (Oliv.) Diels [Apiaceae] (3.0 g), *Bupleurum chinense* DC [Apiaceae] (2.0 g), and *Glycyrrhiza glabra* L [Fabaceae] (1.5 g), were mixed and extracted with purified water (600 ml) at 95.1°C for 1 h. Insoluble waste was separated and concentrated by removing water under reduced pressure. The dried extract contains at least 0.15 mg of total alkaloids, such as rhynchophylline and hirsutine, 0.6–2.4 mg of saikosaponin b2 and 10–30 mg of glycyrrhizic acid. The three-dimensional high-performance liquid chromatography (3D-HPLC) profile chart of YKS was provided by Tsumura and Co. (Figure 1A). YKS powder was dissolved in distilled water and orally administered to the rat at 0.3 or 1.0 g/kg/day after an hour of deprived food (to allow fast assimilation of YKS solution), 30 min before the morphine injection while the control rats received distilled water (10 ml/kg) instead. YKS treatment was initiated 7 days prior to morphine injection; 28 days of YKS pretreatment has been reported to suppress morphine tolerance (Nakagawa et al., 2012), and YKS administration initiated on the day of the first morphine injection has no effect on morphine tolerance development (Takemoto et al., 2016). Therefore, prior to this study, we conducted a preliminary study on the appropriate number of pretreatment days and found that 7 days of pretreatment was effective. The doses of YKS were selected based on previous reports showing beneficial effect of it in morphine tolerance (Nakagawa et al., 2012), aggressive behavior (Nishi et al., 2012; Tabuchi et al., 2017), and anxiety (Ohno et al., 2018) in mice and rats. Morphine solution was prepared by dissolving morphine hydrochloride (T1-02591; Takeda Chemical Industries, Osaka, Japan) in physiological saline (10 mg/kg/day) and was injected subcutaneously 30 min after YKS administration. Physiological saline was injected to the control (CON) and YKS only (YKS) groups.

### Experimental Design

Rats were randomly assigned to one of the following five experimental groups: Control (CON), Morphine, YKS (0.3 g/kg/day) + Morphine (M + 0.3YKS), YKS (1.0 g/kg/day) + Morphine (M + 1.0YKS), and YKS (1.0 g/kg/day) only (YKS). Rats in these groups were orally administered either YKS or vehicle for 12 days and injected subcutaneously morphine or saline daily for the last 5 days (Figure 1B).

### The Hot Plate Test

Expression of tolerance to the analgesic effect of morphine was assessed using the hot plate test 1 hour after morphine injection. The experimental apparatus consisted of a transparent acrylic cylinder (20 cm in diameter and 30 cm in height) fixed to a heating plate maintained at 55 ± 0.2°C during the test (hot-plate apparatus, Ugo Basile, Italy). Rats were individually placed onto the hot plate and the latency to lick, shake, or step on the hind paw was used as an endpoint and was recorded using two video cameras. The cut-off time was set at 45 s to avoid thermal damage to the tissues. Based on our previous studies that showed no difference in the thermal latency on the second day (Takemoto et al., 2016; Katayama et al., 2018), the evaluation of the thermal latency was not performed on the second day.

### Western Blotting

On Day 5, 1 hour after injection of morphine, a spinal cord segment, lumbar vertebrae 5 (L5)—L6, was sampled promptly and quickly frozen with liquid nitrogen. The tissue was homogenized with lysis buffer containing 1% sodium dodecyl sulfate (SDS), 20 mM Tris-HCL (pH 7.4), 5 mM ethylene-diamine-tetraacetic acid (pH 8.0), 10 mM sodium fluoride, 2 mM sodium orthovanadate, 1 mM phenylmethylsulfonyl fluoride, and 0.5 mM phenylarsine oxide. The homogenate was centrifuged at 15,000 rpm for 30 min at 25 °C, and the supernatant was collected. To standardize the sample, the protein concentration was determined using a bicinchoninic acid protein assay kit (Thermo Fisher Scientific, MA, United States). The samples (10 μg protein each) were subjected to sodium dodecyl sulfate polyacrylamide gel electrophoresis (SDS-PAGE, 8 or 10% SDS) and transferred onto a polyvinylidene difluoride membrane. The membrane was blocked with 5% (w/v) BSA (Fujifilm Wako Pure Chemical, Tokyo, Japan) for 1 h at room temperature and then incubated with the following primary antibodies overnight 4°C: The first antibodies used in this study were as follows: anti-syntaxin (1:5,000, #S0664, Sigma-Aldrich Japan Co., Tokyo, Japan); anti-SNAP25 (1:5,000, #111 002, Synaptic Systems, Goettingen, Germany); anti-Synaptotagmin I (1:500, #MAB5200, Millipore); anti-β-actin 1:1,000, #4970, Cell Signaling Technology, Billerica, MA); anti-NR2A (1:500, # GluRe1C-Rb-Af542, Frontier Institute co. ltd., Hokkaido, Japan); anti-NR2B (1:500, GluRe2N-Rb-Af660, Frontier Institute co. ltd.); anti-GluA2/3/4 (1:500, #2460, Cell Signaling Technology); anti-PSD95 (1:500, #MA1-045, Thermo Fisher Scientific); anti-GAD65/67 (1:500, #G5163, Sigma-Aldrich Japan Co., Tokyo, Japan).

The membrane was washed with Tris-buffered saline buffer with Tween 20 (Sigma-Aldrich Japan Co.) and incubated with goat anti-mouse secondary antibody, conjugated with horseradish peroxidase (1:1,000; Jackson ImmunoResearch, PA, United States) or goat anti-rabbit secondary antibody (1:1,000, Rockland Immunochemicals, Gilbertsville, PA, United States) for 2 h at room temperature. Chemiluminescence image was obtained with a Pierce ECL Western blotting substrate (Thermo Fisher Scientific) and captured with a charged-coupled device camera system (Ez-Capture MG, Atto Co., Tokyo, Japan). The intensity of each band was quantified using the Lane and Spot Analyzer software (Atto Co.).

### Immunohistochemistry

Rats were deeply anesthetized 1 h after the last injection of morphine with intraperitoneal administration of a combination of three anesthetics medetomidine hydrochloride, 0.3 mg/kg (Domitol; Nippon Zenyaku Kogyo Co., Ltd., Fukushima, Japan); midazolam, 4.0 mg/kg (Sandoz; Sandoz K.K. Tokyo, Japan) and butorphanol, 5.0 mg/kg (Vetorphale; Meiji Seika Pharma Co., Ltd. Tokyo, Japan) and intracardially perfused with cold phosphate buffered saline (PBS) at pH 7.4, followed by 4% paraformaldehyde in 0.1 M PBS. After perfusion, the spinal cord was removed and post-fixed in the same fixative solution overnight. The spinal cord was equilibrated in 30% sucrose for 2 days and then stored at −80°C. After cutting the tissue into 20 μm thickness using a cryostat (CM 1860; Leica Biosystems, Germany), immunostaining was performed. Sections were rinsed three times in PBS, subsequently incubated with blocking solution (10% goat serum with 0.3% Triton X-100 in PBS), and then incubated with primary antibodies: anti-Neu N (1:500, #MAB377, Millipore, CA, United States), anti-Synaptotagmin I (1:500, #MAB5200, Millipore), anti-VGLUT2 (1:500, #AB2251-I, Millipore) for 48 h. After washing three times with PBS, sections were reacted with AlexaFluor 488 or 546-conjugated secondary antibodies (1:200, Invitrogen, CA, United States). For isolectin B4 (IB4) staining, sections were incubated with isolectin GS-IB4 From *Griffonia simplicifolia*, Alexa Fluor conjugate (1: 500, Invitrogen) instead of primary and secondary antibodies. Sections were subsequently mounted on slides and coverslipped with mounting medium containing DAPI (Santa Cruz Biotechnology, CA, United States). Immunofluorescent images were visualized under Olympus FV1000D confocal microscope (Olympus, Tokyo, Japan). The immunoreactivity of synaptotagmin I and VGLUT2 in the lamina II of the dorsal horn in the spinal cord was measured using an imaging analysis software (FV10-ASW, Olympus). Spinal cord sections obtained from three rats were analyzed for each group.

### Statistical Analysis

All the values are expressed as means and standard error of the mean (SEM). The data was analyzed by one-way ANOVA followed by Bonferroni *post-hoc* test, using SPSS ver. 25 (IBM Japan, Tokyo, Japan) *p*-value < 0.05 was considered statistically significant.

## Results

### Effect of YKS Pretreatment on Morphine Tolerance Formation

**FIGURE 2:**
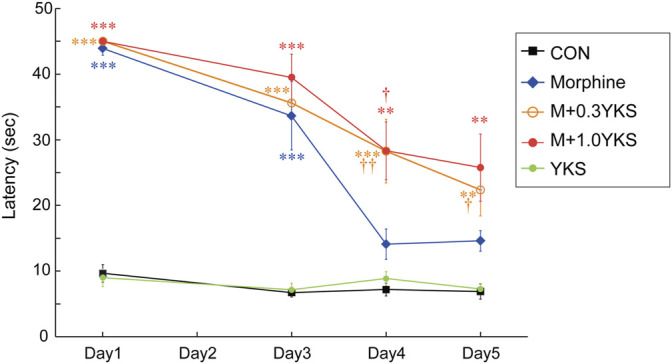
Inhibitory effect of YKS on the development of morphine tolerance in the hot plate test. Morphine tolerance became pronounced on Day 4. YKS significantly suppressed the morphine tolerance at both doses, 0.3 and 1.0 g/kg/day. Values are the mean ± SEM (*n* = 7–9) **p < 0.01, ***p < 0.001 vs. CON group; ^†^ *p* < 0.05, ^††^ *p* < 0.01 vs. Morphine group.

A morphine-tolerant rat model was developed by injecting morphine (10 mg/kg/day) daily for 5 days. YKS (0.3 or 1.0 g/kg/day) administration was started 7 days before the first morphine injection (Figure 1B). On Day 1 (the first day of morphine injection), morphine completely blocked nociception by thermal stimuli in the hot plate test (Day 1: F (4,34) = 78.9, *p* < 0.001) (Figure 2). Pretreatment with YKS for 7 days itself did not show any analgesic effect nor any negative effect on the analgesic effect of morphine on Day 1 (Figure 2). While the morphine antinociceptive tolerance was appeared from Day 4 (Morphine group, Figure 2), the withdrawal latencies of the M+0.3YKS and M+1.0YKS groups were significantly longer than that of Morphine group on Day 4 and 5 (Day 4: F (4,34) = 11.8, *p* < 0.001) (Day 5: F (4,34) = 9.76, *p* < 0.001) (Figure 2), suggesting that administration of YKS suppressed the morphine antinociceptive tolerance.

### YKS Suppressed the Increase in Presynaptic Protein Levels in the Spinal Cord Induced by Chronic Morphine Administration

**FIGURE 3:**
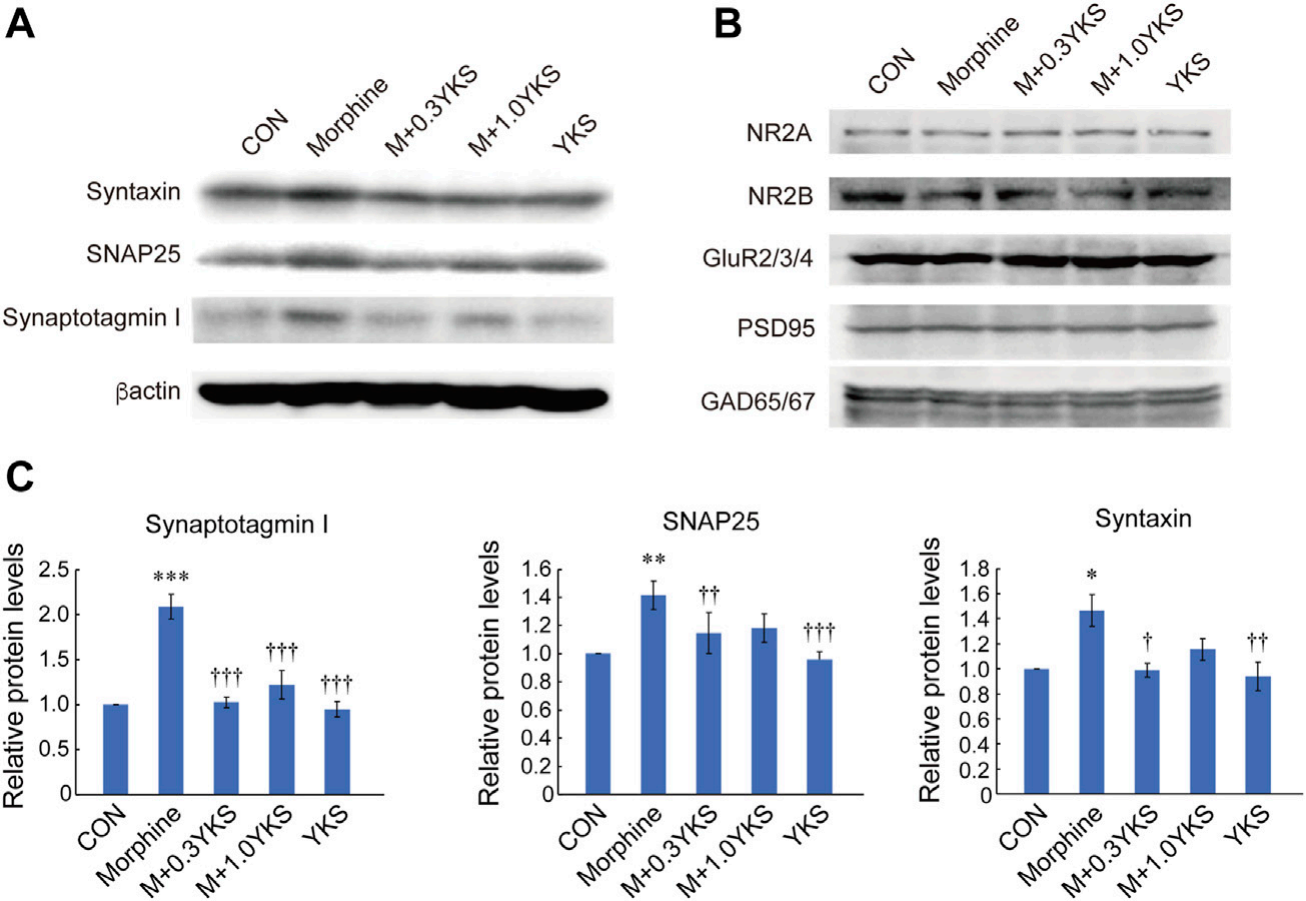
Increased expression of presynaptic proteins in the spinal cord in conjunction with the morphine tolerance formation, which was inhibited by YKS administration. **(A)** Presynaptic protein levels at Day 5 in the spinal cord. **(B)** There were no significant changes in postsynaptic glutamate receptor subunits, PSD 95, and GAD 65/67 protein levels at Day 5. **(C)** Synaptotagmin I, SNAP 25, and syntaxin levels shown in **(A)** were quantified. Values are the mean ± SEM (*n* = 5) *p < 0.05, **p < 0.01, ***p < 0.001 vs. CON group; ^††^ *p* < 0.01, ^†††^ *p* < 0.001 vs. Morphine group.

The expression levels of proteins that function at synapses were measured by western blot analysis in the lumbar vertebrae 5 (L5)—L6 spinal cord collected on Day 5. Interestingly, presynaptic proteins, including synaptotagmin I, SNAP 25, and syntaxin, were significantly increased in the Morphine group. The increase in synaptotagmin I was blocked by administration of YKS at both doses (synaptotagmin I: F (4,20) = 20.9, *p* < 0.001) while 0.3 g/kg/day YKS significantly inhibited its effect on SNAP25 and syntaxin (SNAP 25: F (4,20) = 8.70, *p* < 0.001) (syntaxin: F (4,34) = 6.01, *p* < 0.01) (Figures 3A,C). YKS itself did not affect these protein levels (Figures 3A,C). Unlike presynaptic proteins, the expression levels of postsynaptic proteins, such as NMDA glutamate receptors (NR2A, 2B), AMPA glutamate receptors (GluR 2/3/4), and PSD95, were not altered by either morphine or YKS administration (Figure 3B). Notably, “inhibitory” GABAergic presynaptic marker protein, GAD 65/67, was not altered by both morphine and YKS (Figure 3B). These results indicate that increased levels of glutamatergic presynaptic proteins in the spinal cord are associated with tolerance of antinociception by morphine.

### Chronic Morphine Treatment Enhances Presynaptic Proteins of Glutamatergic DRG Neurons in the L5-L6 Spinal Cord, Which Was Suppressed by YKS

**FIGURE 4:**
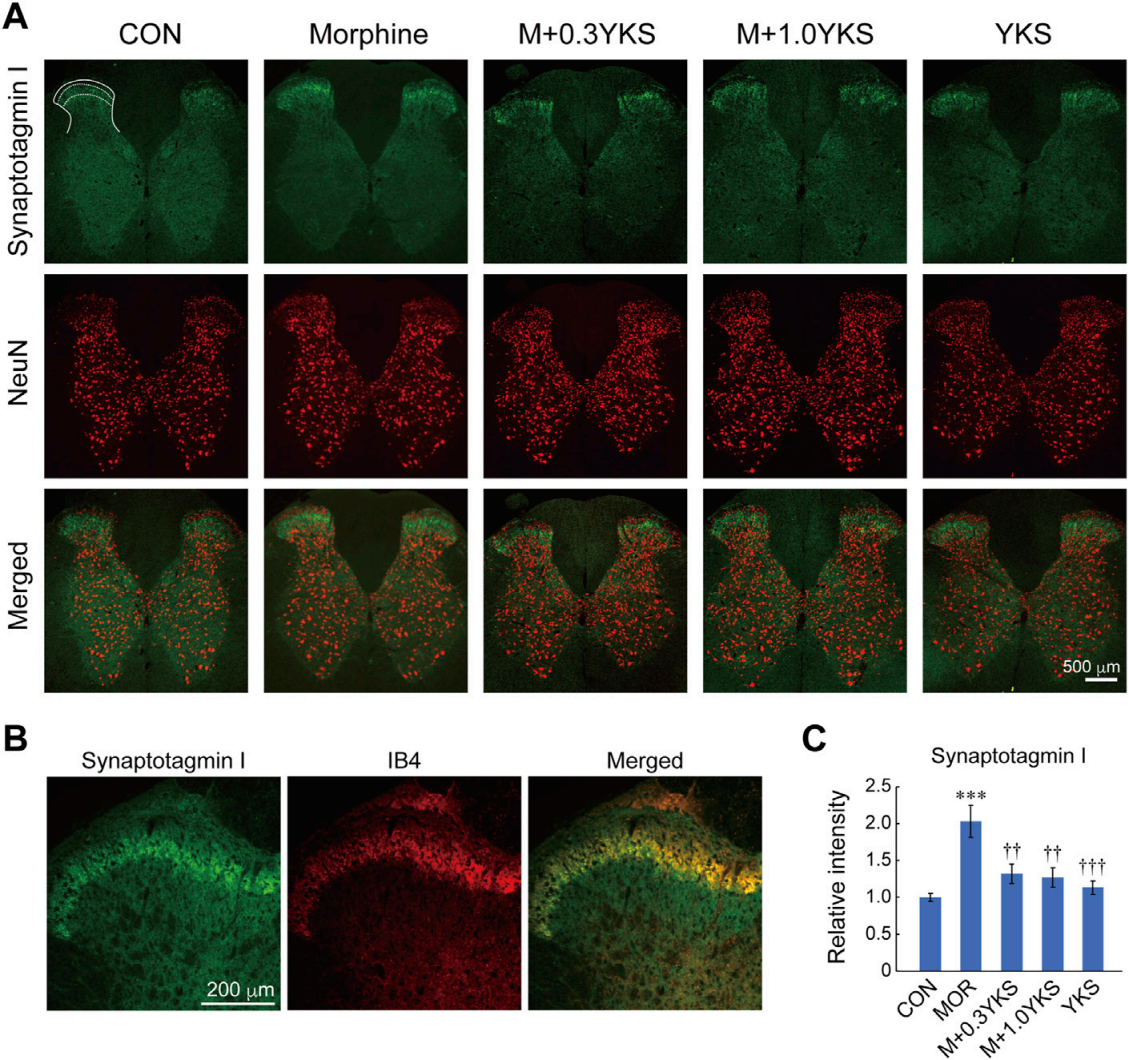
Morphine increased the expression of synaptotagmin I in the lamina II of the spinal cord dorsal horn, and YKS inhibited the increase. **(A)** Synaptotagmin I-positive presynaptic terminals (Green) were observed especially in the lamina II of the dorsal horns. Neural cells were visualized by anti-NeuN antibody (Red). Merged images were also shown. White bar = 500 μm. **(B)** Magnified images for synaptotagmin I and IB4 in the dorsal horn. Green: synaptotagmin I; Red: IB4. White bar = 200 μm. **(C)** Quantified data for the immunoreactivities of synaptotagmin I in lamina II of the dorsal horn. The mean immunoreactivity of synaptotagmin I in each section was determined in the synaptotagmin I-expressing lamina II region [The area bounded by two dashed and solid lines in (A)]. The spinal cord sections were obtained from three rats in each group. The number of (sections and dorsal horns) determined in this experiment were: CON (16 and 32); Morphine (17 and 33); M+0.3YKS (15 and 29); M+1.0YKS (16 and 30); YKS (23 and 42). ***p < 0.001 vs CON group; ^††^ *p* < 0.01, ^†††^ *p* < 0.001 vs Morphine group.

**FIGURE 5:**
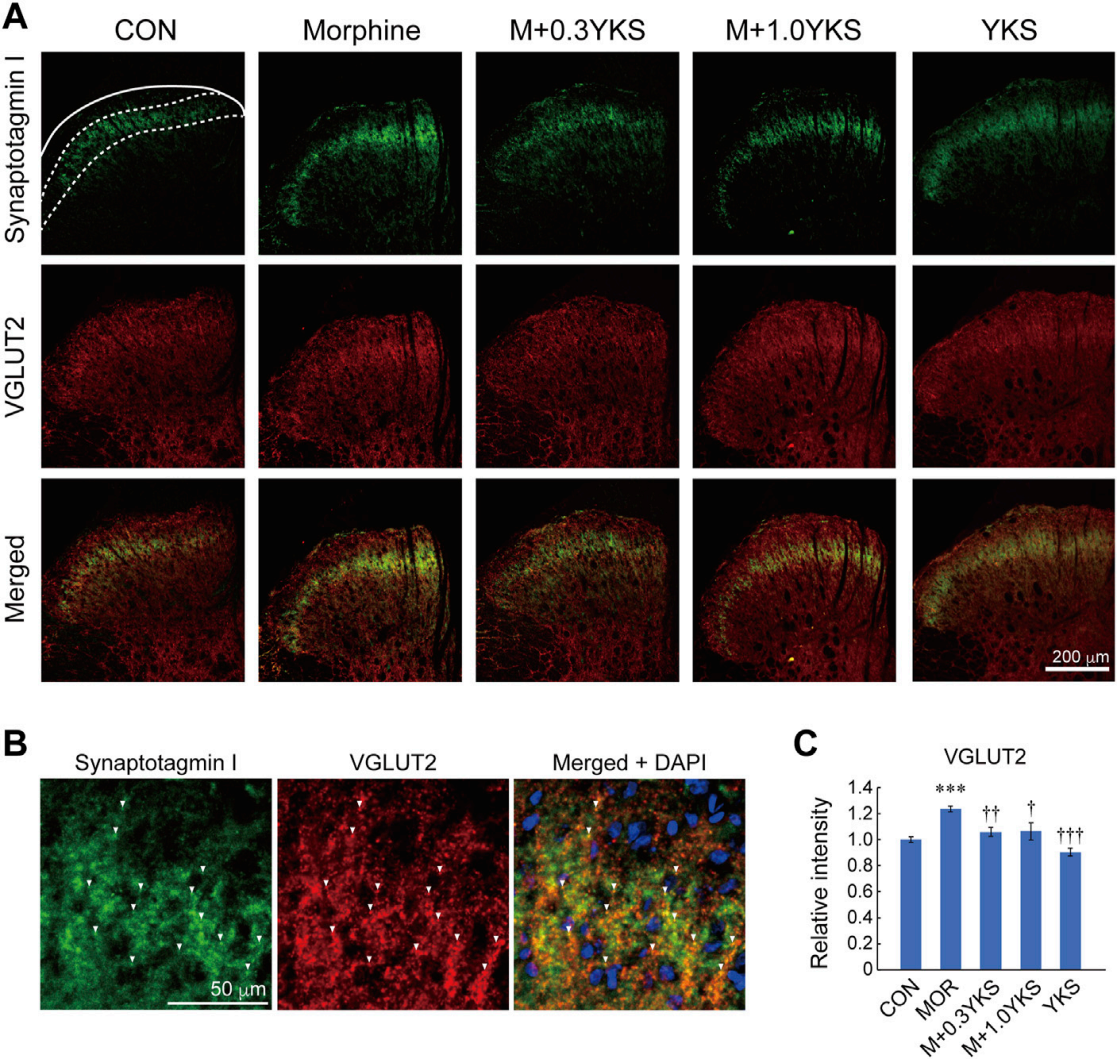
Morphine increased the expression of VGLUT2 in lamina II of the spinal cord dorsal horn, which was inhibited by YKS. **(A)** Immunostaining images for synaptotagmin I and VGLUT2 in the dorsal horn. Green: synaptotagmin I; Red: VGLUT2. White bar = 200 μm. **(B)** Magnified images for synaptotagmin I and VGLUT2 in lamina II. Green: synaptotagmin I; Red: VGLUT2. White bar = 50 μm. White arrow heads indicate the representative synaptotagmin I-positive presynaptic terminals that expressed VGLUT2. **(C)** Quantified data for VGLUT2 in the synaptotagmin I-expressing region. The mean immunoreactivity of VGLUT2 in each section was determined in the synaptotagmin I-expressing lamina II region [The area bounded by two dashed and solid line in (A)]. The spinal cord sections were obtained from three rats in each group. The number of (sections and dorsal horns) determined in this experiment were: CON (18 and 36); Morphine (18 and 36); M+0.3YKS (19 and 38); M+1.0YKS (13 and 24); YKS (20 and 40). ***p < 0.001 vs.CON group; ^†^ *p* < 0.05, ^††^ *p* < 0.01, ^†††^ *p* < 0.001 vs. Morphine group.

To assess which regions of the spinal cord were involved in the morphine-induced enhancement of presynaptic protein levels, sections of the L5-L6 spinal cord were immunostained with antibodies against presynaptic proteins. We found with surprise that synaptotagmin I was predominantly expressed around the lamina II of the spinal dorsal horn where receives sensory input from DRG neurons (Figure 4A). Interestingly, synaptotagmin I-positive presynaptic terminals around the lamina II was well colocalized with IB4-positive region (Figure 4B), suggesting that synaptotagmin I expression observed in the lamina II of the dorsal horn could be mainly attributed to IB4-positive DRG neurons. Morphine treatment increased immunoreactivity of synaptotagmin I in the region (synaptotagmin I: F (4,161) = 8.84, *p* < 0.001), as revealed by western blotting (Figures 4A,C). Pretreatment and co-administration of YKS along with morphine injection prevented or suppressed the enhancement of synaptotagmin I expression induced by morphine (Figures 4A,C). Vesicular glutamate transporter 2 (VGLUT2), which transports glutamate into synaptic vesicles and is used as a marker of presynaptic terminals of glutamatergic neurons, was also strongly expressed in the lamina II (Figure 5A). Substantial part of synaptotagmin I-positive presynaptic terminals expressed VGLUT2 (Figure 5B). Morphine slightly but significantly enhanced its expression in the synaptotagmin I-expressing region (VGLUT2_lamina II: F (4,169) = 14.1, *p* < 0.001) (Figures 5A,C) while VGLUT2 immunoreactivity in the anterior horn was not changed by morphine nor YKS treatment (VGLUT2_anterior horn: F (4,169) = 0.759, *p* = 0.554) (data not shown). The morphine-induced increase in VGLUT2 levels in the synaptotagmin I-expressing region was also inhibited by YKS administration (Figures 5A,C). These data indicate that the enhancement of presynaptic functions of IB4-positve glutamatergic DRG neurons is involved in the morphine tolerance, and YKS inhibited it through suppression of the morphine-induced enhancement of presynaptic protein expression levels.

## Discussion

**FIGURE 6:**
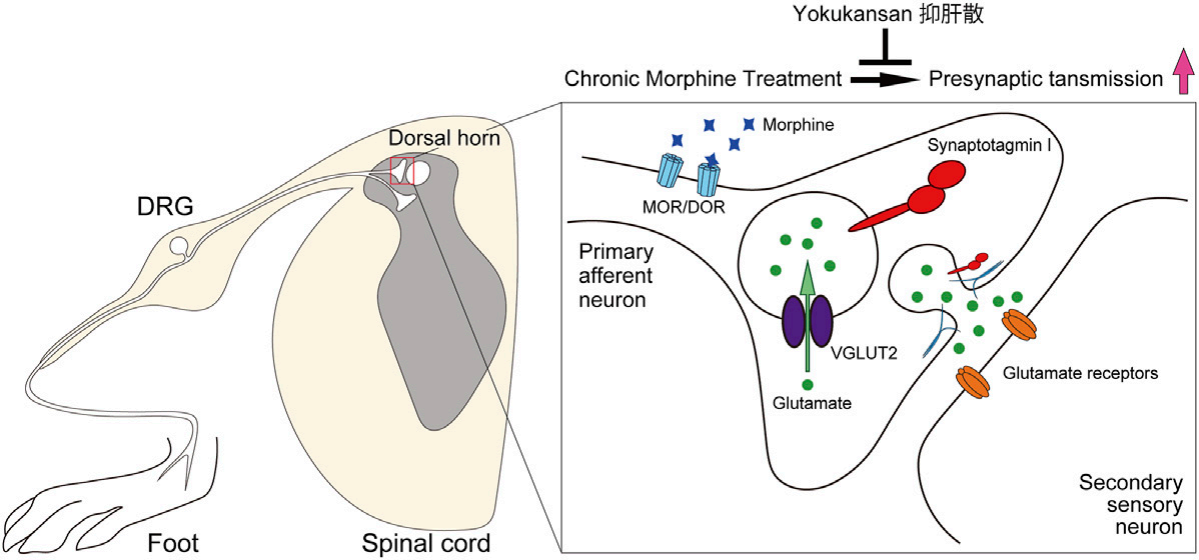
Schematic illustration of the effect of YKS on the development of morphine antinociceptive tolerance. Continuous morphine administration would enhance of glutamatergic synaptic transmission between DRG neurons and the secondary neurons in the spinal cord dorsal horn *via* increasing presynaptic proteins without affecting postsynaptic functions during morphine tolerance formation. Pretreatment of YKS suppressed the potentiation and inhibited the development of morphine tolerance. DRG: dorsal root ganglion, MOR: μ-opioid receptor. DOR: δ-opioid receptor.

In the present study, we have elucidated novel mechanisms for the morphine tolerance formation and the suppressive effect of YKS in the morphine tolerance. Our results suggest that the enhancement of excitatory synaptic transmission between primary sensory DRG neurons and secondary neurons in the dorsal horn is involved in the development of morphine tolerance (Figure 6). Morphine would act on DRG neurons to enhance presynaptic function without affecting postsynaptic proteins. YKS administration may inhibit the development of morphine tolerance *via* suppressing the hyperfunction of the presynaptic transmission of DRG neurons (Figure 6). Our findings strongly support that presynaptic protein level has an intrinsic role in the development of morphine antinociceptive tolerance.

We firstly performed the hot plate test to examine the development of morphine antinociceptive tolerance in different groups of rats. The choice of this test was based on its rapidity and sensitivity in screening for analgesic effects. As expected, rats in the morphine group showed morphine tolerance from Day 4 of morphine injection, as indicated by a significant decrease in response to thermal stimulation. However, two doses of YKS altered the expression of this behavior. These data are consistent with our previous studies (Takemoto et al., 2016; Katayama et al., 2018), which showed that repeated YKS pretreatment suppressed the development of morphine tolerance.

It has been demonstrated that DRG neuron-specific MOR knockout in mice abolishes the analgesic effect of morphine and opioid-induced hyperalgesia (Corder et al., 2017; Sun et al., 2020). Deletion of MOR specifically in TRPV1- (Corder et al., 2017) or Advillin-expressing DRG neurons (Sun et al., 2019) almost completely abolished the analgesic effect by acute treatment of MOR agonist or hyperalgesia caused by chronic opioid treatment. Sun et al. reported in the electrophysiology studies that the analgesic effect of morphine is largely due to the inhibitory effects on glutamatergic transmission by DRG neurons to the secondary neurons in the spinal cord dorsal horn via MOR in DRG neurons (Sun et al., 2019; 2020). They further showed that stimulatory effects of MOR agonist on glutamatergic transmission to spinal dorsal horn neurons occurred after the inhibitory effects, which would be essential for the development of morphine tolerance (Sun et al., 2019; 2020). These data clearly suggests that plastic changes in the presynaptic function of the MOR-expressing DRG neurons are mainly involved in the formation of morphine tolerance.

The increased immunoreactivity of synaptotagmin I around the lamina II in the dorsal horn during the morphine tolerance formation and its inhibition by YKS suggest that the regulation of synaptic transmission efficacy between DRG neurons and secondary neurons in the dorsal horn would be important for the development of morphine tolerance and the effect of YKS. We unexpectedly found that not all but a significant number of synaptotagmin I-positive presynaptic terminals were IB4-positive. The majority of IB4-positive DRG neurons are small, have unmyelinated afferents, and are classically classified into non-peptidergic sensory neurons, divided from peptidergic ones releasing neuropeptides such as calcitonin gene-related peptide (CGRP) (Marmigère and Ernfors, 2007). Peptidergic sensory neurons had been thought as the main fibers transmitting thermal and mechanical nociceptive stimuli. However recent studies have revealed an important function of non-peptidergic sensory neurons in thermal nociception. Up to 70% of IB4-positive sensory neurons expressed TRPV1 (Cui et al., 2014) and selective ablation of IB4-positive DRG neurons increased thermal nociceptive threshold (Vulchanova et al., 2001) in rats. Consistent with previous study showing that about 80% of IB4-positive DRG neurons expressed VGLUT2 (Scherrer et al., 2010), immunoreactivity of VGLUT2 in the dorsal horn was well correlated with that of synaptotagmin I, indicating the importance of presynaptic potentiation in glutamatergic synapses between DRG neurons and secondary neurons in morphine tolerance formation. These data strongly suggests that IB4-positive DRG neurons would be one of the points of the action of YKS. Furthermore, the involvement of “glutamatergic” synapse transmission in the spinal cord in the morphine tolerance formation was also supported by the stable expression of GAD 65/67, a marker for “inhibitory” GABAergic presynaptic terminals, after both morphine and YKS treatment (Figure 3B). These studies including ours suggest that chronic treatment of morphine could potentiate glutamate transmission from DRG neurons to the secondary sensory neurons in the dorsal hone, which could be suppressed by YKS pretreatment. IB4-positive DRG neurons would contribute to the development of morphine tolerance and the YKS effect.

In the present study, YKS itself had no antinociceptive effect, suggesting that YKS has no agonist activity on MOR or its downstream intracellular signals involved in the acute depression of glutamatergic transmission, but suppresses the delayed synaptic potentiation caused by chronic morphine treatment.

Some studies investigated the individual contribution of NMDA and AMPA glutamate receptors subunits in morphine tolerance, for example, Xiao et al. documented an increase in NR1 (GluN1) and NR2B (GluN2B), NMDA receptor subunits, expression after 7-days morphine treatment (Xiao et al., 2019). Furthermore, Huang et al. found that chronic morphine treatment increased NR2A expression and phosphorylated NR2B in the post synaptic density (PSD) fractions (Huang et al., 2019). These studies are conflicting with our results where no changes in postsynaptic protein expressions, including NR2A (GluN2A) and NR2B expression, have been observed in the spinal cord. This discrepancy is probably due to intrathecal morphine administration used in these two studies, which might affect secondary sensory neurons in the spinal cord more directly than subcutaneous administration used in our study and suggests that postsynaptic changes in the secondary sensory neurons in the spinal cord might secondarily occur later in time than the presynaptic changes in DRG neurons. However, changes in the expression of postsynaptic proteins due to YKS function need to be examined in the PSD fraction in the future.

Several studies have reported that chronic morphine treatment can activate glial cells in the spinal cord, which leads to the release of cytokines, such as interleukin-1β (IL-1β), IL-6, tumor necrosis factor-α (TNF-α), and of inflammatory immune response in L5 lumbar spinal cord, resulting in the development of morphine tolerance (Song and Zhao 2001; Raghavendra et al., 2004). Meanwhile, it was reported that MOR mRNA is expressed exclusively in neurons but not in astrocytes or microglia in the spinal cord (Kao et al., 2012), which suggests that morphine-induced glial cell activation in the spinal cord is secondary to the enhancement of synaptic transmission in DRG neurons (Corder et al., 2017). We also previously reported that YKS suppresses morphine-induced glial cell activation in the spinal cord (Takemoto et al., 2016). Taken together with the present study, YKS may improve morphine tolerance through multiple pathways through which YKS components act.

Although the mechanism of the presynaptic potentiation of glutamatergic transmission in DRG neurons by chronic treatment of morphine remains unknown, the results of the present study indicate that an increase in glutamate-containing synaptic vesicles in the terminal of DRG neurons or an increase in the number of presynaptic sites on the secondary sensory neurons in the dorsal horn might at least partially contribute it. YKS had an inhibitory effect on this presynaptic glutamatergic potentiation in this study. Although the pathogenesis is different and the mechanism of action is unclear, Takeda and colleagues also reported that YKS suppressed the abnormal increase in extracellular glutamate in rats fed a zinc-deficient diet (Takeda et al., 2008). Furthermore, several components of YKS, such as 18-β-glycyrrhetinic acid (Tabuchi et al., 2012), liquiritigenin (Li et al., 2015), and geissoschizine methyl ether (Imamura et al., 2011; Matsumoto et al., 2020) which have been implicated in modulating pain and glutamate toxicity, have been shown to penetrate the blood-brain barrier (BBB). It will be important to elucidate the detailed mechanism of the YKS action on glutamatergic transmission in DRG neurons and which ingredients in YKS act on them. Morphine acts primarily through MOR, but there is evidence that the δ-opioid receptor (DOR) is critical for the development of morphine antinociceptive tolerance (Xie et al., 2009). DOR knockout mice does not developed morphine tolerance (Zhu et al., 1999; Nitsche et al., 2002) and activation of DOR has been shown to be a critical step for the development of tolerance (Xie et al., 2009). Importantly, IB4-positive DRG neurons have distinct DOR expression and about 37% of them also express MOR (Yamamoto et al., 2008). Altogether, the present study shows that potentiation in glutamate transmission of IB4-positive DRG neurons may play a substantial role in the development of morphine tolerance and YKS may block it by acting on IB4-positive sensory neurons although a limitation of this study is that there has been no direct analysis of the effects of morphine or YKS in the DRG. Future studies that include such an analysis would provide a more detailed mechanism for morphine tolerance formation and the effect of YKS on it.

Herbal medicines such as Kampo medicine have been reluctant to be used in clinical practice from the viewpoint of evidence-based medicine. However recent basic researches have revealed many beneficial effects of herbal medicines and their molecular mechanisms. As for morphine tolerance, YKS suppressed not only synaptic potentiation of DRG neurons shown in this study, but also glial activation in the spinal cord (Takemoto et al., 2016) and orexin secretion in the midbrain (Katayama et al., 2018). Because of the advantages of herbal medicines in terms of fewer side effects and combined action, it is highly expected that the use of YKS in combination with opioids for pain management will increase in clinical practice in the future.

## Data Availability

The raw data supporting the conclusion of this article will be made available by the authors, without undue reservation.
